# *Klebsiella pneumoniae* ST307 with *bla*_OXA-181,_ South Africa, 2014–2016

**DOI:** 10.3201/eid2504.181482

**Published:** 2019-04

**Authors:** Michelle Lowe, Marleen M. Kock, Jennifer Coetzee, Ebrahim Hoosien, Gisele Peirano, Kathy-Ann Strydom, Marthie M. Ehlers, Nontombi M. Mbelle, Elena Shashkina, David B. Haslam, Puneet Dhawan, Robert J. Donnelly, Liang Chen, Barry N. Kreiswirth, Johann D.D. Pitout

**Affiliations:** University of Pretoria, Pretoria, South Africa (M. Lowe, M.M. Kock, K.-A. Strydom, M.M. Ehlers, N.M. Mbelle, J.D.D. Pitout);; National Health Laboratory Service, Pretoria (M. Lowe, M.M. Kock, K.-A. Strydom, M.M. Ehlers, N.M. Mbelle);; Ampath Laboratories, Pretoria (J. Coetzee, E. Hoosien);; Calgary Laboratory Services, Calgary, Alberta, Canada (G. Peirano, J.D.D. Pitout);; University of Calgary, Calgary (G. Peirano, J.D.D. Pitout);; Rutgers University, Newark, New Jersey, USA (E. Shashkina, L. Chen, B.N. Kreiswirth);; Cincinnati Children's Hospital Medical Center, Cincinnati, Ohio, USA (D.B. Haslam);; New Jersey Medical School, Newark (P. Dhawan, R.J. Donnelly)

**Keywords:** *Klebsiella pneumoniae*, ST307, carbapenemases, *Enterobacteriaceae*, genomic surveillance, outbreak, antimicrobial resistance, bacteria, South Africa, sequence type

## Abstract

*Klebsiella pneumoniae* sequence type (ST) 307 is an emerging global antimicrobial drug–resistant clone. We used whole-genome sequencing and PCR to characterize *K. pneumoniae* ST307 with oxacillinase (OXA) 181 carbapenemase across several private hospitals in South Africa during 2014–2016. The South Africa ST307 belonged to a different clade (clade VI) with unique genomic characteristics when compared with global ST307 (clades I–V). Bayesian evolution analysis showed that clade VI emerged around March 2013 in Gauteng Province, South Africa, and then evolved during 2014 into 2 distinct lineages. *K. pneumoniae* ST307 clade VI with OXA-181 disseminated over a 15-month period within 42 hospitals in 23 cities across 6 northeastern provinces, affecting 350 patients. The rapid expansion of ST307 was most likely due to intrahospital, interhospital, intercity, and interprovince movements of patients. This study highlights the importance of molecular surveillance for tracking emerging antimicrobial clones.

The World Health Organization recently identified global spread of antimicrobial resistance (AMR) as one of the most serious recent threats to human health (*1*). The emergence and spread of carbapenem resistance is a substantial public health concern, because these agents are regarded as one of the last effective therapies available for treating serious infections caused by gram-negative bacteria. Carbapenemases, the predominant cause of carbapenem resistance, are commonly harbored on plasmids that are able to transfer between members of the family *Enterobacteriaceae* (*2*). The most common carbapenemases among clinical *Enterobacteriaceae* are the *Klebsiella pneumoniae* carbapenemases (KPCs; Ambler class A); the metallo-β-lactamases (IMPs, VIMs, NDMs; Ambler class B); and oxacillinase 48 (OXA-48)–like (Ambler class D) enzymes.

The OXA-48–like carbapenemases are identified mostly in *K. pneumoniae* and *Escherichia coli* and include the following enzymes: OXA-48, OXA-162, OXA-181, OXA-204, OXA-232, OXA-244, OXA-245, and OXA-247 (*3*). They are active against penicillins and weakly hydrolyze carbapenems, with limited activities against broad-spectrum cephalosporins and most β-lactam inhibitors.

The earliest reported case in South Africa of a *K. pneumoniae* that contained *bla*_OXA-48_ occurred in Johannesburg in 2011; the case-patient had previously been hospitalized in Egypt (*4*). The report also described OXA-181–producing *K. pneumoniae* from different Johannesburg and Cape Town private hospitals. Laboratory surveillance reports showed that OXA-48–like enzymes are the second most common carbapenemase (after NDMs) in various healthcare centers across South Africa (*5*).

The molecular diagnostic reference center at Ampath Laboratories (Ampath-MDRC) in Pretoria, Gauteng Province, South Africa, experienced a substantial increase of *K. pneumoniae* with *bla*_OXA-48-like_ during 2014–2016 from different private hospitals across northeastern South Africa. We designed a study to investigate the underlying molecular mechanisms associated with the increase of *K. pneumoniae* with OXA-48–like enzymes. We obtained ethics approval from the Conjoint Health Research Ethics Board at the University of Calgary (REB17-1010) and from the Research Ethics Committee (REC), Faculty of Health Sciences, University of Pretoria (protocol nos. 240/2016 and 104/2017).

## Materials and Methods

### Setting and Workflow

We described South Africa’s health system and the role of Ampath-MDRC (Appendix). We also summarized the study workflow (Appendix Figure 1).

We screened all carbapenem-nonsusceptible *K. pneumoniae* at Ampath-MDRC using a PCR for *bla*_NDM_, *bla*_KPC_, *bla*_OXA-48_–like, *bla*_IMP_, *bla*_VIM_, and *bla*_GES_. We performed pulsed-field gel electrophoresis (PFGE) on 471 *K. pneumoniae* isolates positive for OXA-48–like enzyme to determine if some of the isolates were genetically related. We identified 1 dominant pulsotype (pulsotype A) and a related pulsotype (AR) and selected isolates representing them from different geographic sites over various time periods. We used these isolates for initial Illumina short-read sequencing (n = 28) (Illumina, http://www.illumina.com) and PacBio long-read sequencing (n = 1) (Pacific Biosciences, http://www.pacb.com) to determine if they belong to the same sequence type and to identify the OXA-48–like enzyme. 

We identified the pulsotypes as sequence type (ST) 307 containing OXA-181 on IncX3 plasmids. We then compared this whole-genome sequencing (WGS) data with the sequences from the US National Center for Biotechnology Information (NCBI) genome database (ftp://ftp.ncbi.nih.gov/genomes) to design PCR primers for the detection of ST307, IncX3, and OXA-181-IS3000 mobile genetic element (MGE). We used previously characterized *K. pneumoniae* STs with different plasmid replicons to verify these primers (Appendix Table 1). We screened *K. pneumoniae* producing OXA-48–like enzyme (n = 471) with PCR primers to identify ST307, IncX3 plasmids, and OXA-181-IS3000 MGE. We selected additional *K. pneumoniae* ST307 isolates (n = 60) from different geographic locations, time points, and specimens to undergo Illumina short-read WGS to elucidate the evolution of ST307 in South Africa. 

### Bacterial Isolates

During January 2014–December 2016, various Ampath regional clinical laboratories referred 1,247 unique clinical, carbapenem (i.e., ertapenem, meropenem, or imipenem) nonsusceptible *K. pneumoniae* isolates to Ampath-MDRC for PCR confirmation of carbapenemases (Appendix Figure 1). We performed PCR screening for *bla*_NDM_, *bla*_KPC_, *bla*_OXA-48_–like, *bla*_IMP_, *bla*_VIM_, and *bla*_GES_ using LightMix modular carbapenemase kits (TIB Molbiol, https://www.tib-molbiol.com) on a LightCycler 480 II instrument (Roche Diagnostics, https://www.roche.com). Details on the identification and susceptibility testing of the bacterial isolates are provided in the Appendix.

### PFGE

We performed PFGE on the *K. pneumoniae* isolates with OXA-48–like enzymes (n = 471) to determine if there was a dominant pulsotype among them. The Appendix describes the methodology used and results obtained with PFGE.

### PCR 

We designed 3 sets of PCR primers for the detection of ST307, IncX3 plasmid, and the IS3000-OXA MGE. We screened *K. pneumoniae* isolates with OXA-48–like enzymes (n = 471) using different primer sequences (Appendix Table 1).

### WGS and Data Analysis

We sequenced the *K. pneumoniae* isolates that tested positive by PCR for ST307 (n = 88) using the Illumina NextSeq platform (Appendix). We prepared libraries with the Illumina Nextera XT kit to produce paired end reads of 150 bp for a predicted coverage of >75×. We chose *K. pneumoniae* I72 (isolated from urine obtained in Benoni, Gauteng Province, during December 2014) that was positive for *bla*_OXA-181_ and *bla*_OXA-48_ for long-read WGS using the RSII platform (Pacific Biosciences) to characterize plasmids.

We compared the South Africa genomes sequenced in this study (deposited in the NCBI Bioproject database [https://www.ncbi.nlm.nih.gov/bioproject] under accession no. PRJNA488070) with 620 ST307 genomes previously deposited in the NCBI Sequence Read Archive (https://www.ncbi.nlm.nih.gov/sra) and the genome database. The global ST307 genomes were from the United States (n = 488), the United Kingdom (n = 45), Norway (n = 30), Italy (n = 10), Thailand (n = 9), Australia (n = 6), Brazil (n = 3), Colombia (n = 3), China (n = 3), Nepal (n = 3), Cambodia (n = 2), France (n = 2), Nigeria (n = 2), Cameroon (n = 1), Guinea (n = 1), Iran (n = 1), Netherlands (n = 1), Pakistan (n = 1), and other unspecified regions (n = 9).

We mapped all genomes to the ST307 reference genome NR5632 (GenBank accession no. CP025143) using Snippy (https://github.com/tseemann/snippy). We predicted prophages using PHASTER (*6*), examined repeated regions using MUMmer (*7*), and predicted putative regions of recombination with Gubbins (*8*), followed by filtering using vcftools. We generated a recombination-free single-nucleotide polymorphism phylogenetic tree, using a general time-reversible model of nucleotide substitution with a gamma model of rate heterogeneity and 4 rate categories, by using RAxML version 8.2.4 (*9*). We conducted hierarchical Bayesian analysis of population structure with 3 nested levels and 10 independent runs of the stochastic optimization algorithm with the a priori upper bound of 10–30 clusters varying across the runs to identify phylogenetic clades. We defined clades using the first level of clustering (*10*) and annotated the phylogenetic tree in iTOL (*11*).

We used BEAST version 2.4.7 (*12*) to estimate a timed phylogeny with concatenated recombination-free core single-nucleotide polymorphism alignment. To increase the accuracy of the time to most recent common ancestor, we included 17 additional ST307 isolates from a Pretoria academic hospital in this analysis. We identified the *bla*_OXA181_ harboring IncX3 plasmids by de novo assembly using plasmidSPAdes (*13*) and manually investigated the findings using Bandage assembly graph viewer (*14*) and blastn (https://blast.ncbi.nlm.nih.gov/Blast.cgi), as well as S1-PFGE in combination with Southern blotting (Appendix). 

## Results

### Overview 

Ampath-MDRC experienced an increase in OXA-48–like *K. pneumoniae* isolates during July 2015–June 2016, especially from private hospitals in Gauteng Province. We identified by PFGE a dominant pulsotype named A (and a related pulsotype AR) among the OXA-48–like collection. Initial WGS on 28 isolates that belong to pulsotypes A and AR identified them as ST307 with OXA-181 on IncX3 plasmid that was shown using PacBio long-read sequencing. Using WGS, we used PCR primers specific to ST307, IncX3, and OXA-181-IS3000 MGE to screen the OXA-48–like *K. pneumoniae* and showed that 74% belonged to ST307 containing IncX3 plasmids.

WGS of an additional 60 PCR-positive ST307 illustrated, when compared with ST307 from other countries, that the South Africa ST307 belonged to a different clade. The South Africa clade emerged around March 2013 in Gauteng Province, then evolved during 2014 into 2 distinct lineages that spread across northeastern South Africa, affecting 350 patients.

### Increase of OXA-48–Like Positive *K. pneumoniae* across Northeastern South Africa 

Overall, 574 carbapenemase gene PCR-positive *K. pneumoniae* were detected at Ampath-MDRC during January 2014–December 2016; the total included NDM (n = 58), KPC (n = 10), VIM (n = 35), and OXA-48–like (n = 471) carbapenemases (Appendix Table 2). The OXA-48–like isolates from 2014 came mainly from different private hospitals in Johannesburg and Alberton, Gauteng Province. The numbers of OXA-48–like *K. pneumoniae* increased exponentially toward the end of 2015 and peaked during the first 6 months of 2016 (e.g., we detected 349/471 [74%] of OXA-48–like *K. pneumoniae* during July 2015–June 2016). This increase was especially evident in various hospitals across the province. Private hospitals from other areas in northern and eastern South Africa also experienced increases during 2015–2016 (Appendix Table 2). Throughout the study period, the numbers of *K. pneumoniae* with KPC, NDM, and VIM remained relatively low and stable compared with OXA-48–like *K. pneumoniae*. PCR testing did not detect IMP or GES during this period.

We obtained OXA-48–like *K. pneumoniae* from intraabdominal specimens (n = 78), urine (n = 196), skin and soft tissues (n = 11), blood (n = 76), central venous catheter tips (n = 8), respiratory specimens (n = 99), and rectal specimens (n = 3). The isolates tested nonsusceptible (intermediate or full resistance) to ampicillin, amoxicillin/clavulanic acid, piperacillin/tazobactam, cefotaxime, ceftazidime, cefepime, and ertapenem. Most isolates were also nonsusceptible to trimethoprim/sulfamethoxazole (98%), gentamicin (98%), ciprofloxacin (92%), and meropenem (52%), whereas 44% of isolates were nonsusceptible to imipenem and 11% to amikacin. The tigecycline (TGC) and colistin MICs were each <1 µg/mL, except for 4 isolates that had TGC MICs of 2, 4, 4, and 8 µg/mL.

### Dissemination of ST307 across Private Hospitals 

**Figure 1:**
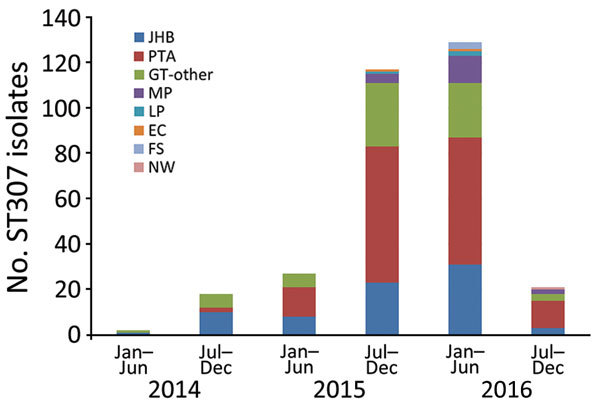
Locations of *Klebsiella pneumoniae* with ST307 oxacillinase 48–like in South Africa during January 2014–December 2016, as identified at Ampath Molecular Diagnostic Reference Center (PTA). EC, Eastern Cape Province; FS, Free State Province; GT-other, other cities in Gauteng Province; JHB, Johannesburg, Gauteng Province; LP, Limpopo Province; MP, Mpumalanga Province; NW, North West Province; PTA, Pretoria, Gauteng Province; ST, sequence type.

**Figure 2:**
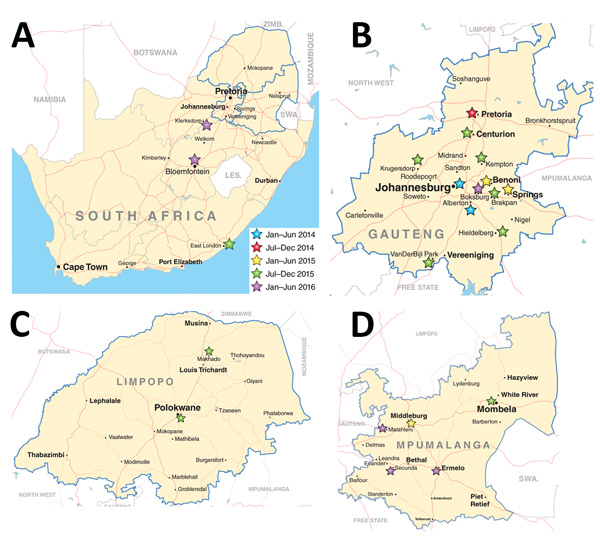
Geographic distribution of *Klebsiella pneumoniae* sequence type 307 with oxacillinase 181 in northeastern South Africa, January 2014–December 2016. A) South Africa; B) Gauteng Province; C) Limpopo Province; D) Mpumalanga Province. Map source: http://d-maps.com.

PCR screening of OXA-48–like *K. pneumoniae* showed that 350/471 (74%) isolates were positive for ST307, IncX3, and IS3000-OXA-181 (cited as ST307_X3-OXA-181). ST307_X3-OXA-181 appeared in different private hospitals in Johannesburg and Alberton during January–June 2014 (Figures 1, 2). During July 2014–June 2016, ST307_X3-OXA-181 subsequently spread to other private hospitals in Gauteng Province, especially in Pretoria, and to Mpumalanga, North West, Limpopo, Free State, and Eastern Cape Provinces (Figures 1, 2). Gauteng Province was the epicenter of the dissemination of ST307_X3-OXA-181, showing a substantial increase in numbers from 2014 (n = 18) to 2015 (n = 138) (Figure 1).

We isolated ST307_X3-OXA-181 mostly from urine (41%), respiratory (20%), intraabdominal (18%), and blood (16%) specimens; 96% of specimens were submitted from hospital settings. Ampath-MDRC receives ≈80% of community, nursing home, and hospital specimens from the private sector in Gauteng, Mpumalanga, North West, Limpopo, Free State, and Eastern Cape Provinces. We isolated ST307_X3-OXA-181 exclusively from hospital specimens; therefore, it is unlikely that the community or nursing home sectors were important reservoirs of ST307.

### South Africa *K. pneumoniae* ST307 Clade

**Figure 3:**
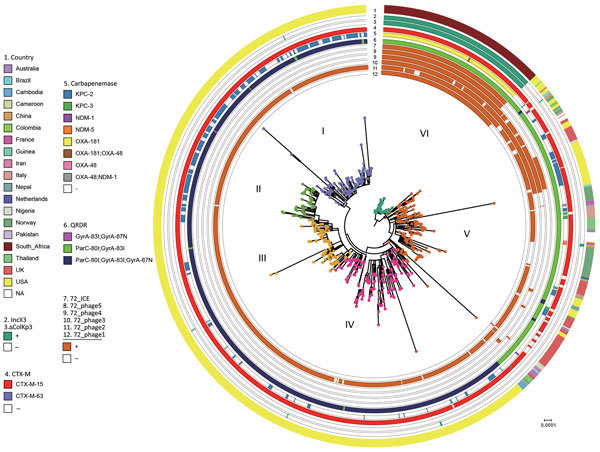
Bayesian phylogenetic analysis of global *Klebsiella pneumoniae* sequence type (ST) 307 isolates. The ST307 genomes included 88 from South Africa (this study) and 620 international isolates from 19 countries (downloaded from the US National Center for Biotechnology Information whole genome shotgun database). ST307 has 6 distinct clades, as indicated on branches. CTX-M, active on cefotaxime first isolated in Munich; KPC, *Klebsiella pneumoniae* carbapenemase; NDM, New Delhi metallo-β-lactamases; OXA, active on oxacillin; QRDR, quinolone resistance determinants.

The overall characteristics of the ST307 sequences from this study (n = 88) were similar to ST307 with *bla*_KPC_ and *bla*_CTX-M-15_ sequences obtained previously in Colombia, Italy, and the United Kingdom, and those in this study contained the capsular loci *wzi*-173, capsule 2, π-fimbrial cluster, Type IV secretion system (*15*). The hierarchical Bayesian clustering analysis of 88 South Africa ST307 and additional 620 genomes from publicly available databases divided ST307 into 6 distinct clades: clades I–IV, from the United States (mainly Texas) (*16*); clade V, from various countries (Australia, Brazil, Cambodia, Cameroon, China, Colombia, France, Guinea, Iran, Italy, Nepal, Netherlands, Nigeria, Norway, Pakistan, Thailand, the United Kingdom, and the United States); and clade VI, which consisted of the isolates from South Africa (Figure 3). Clade V also includes a subset of isolates from Texas. Sequence analysis of the mutations in the quinolone resistance–determining regions in chromosomal *gyrA* and *parC* indicated that most of the isolates from clades V and VI contained the ParC-80I and GyrA-83I mutations, whereas isolates from clades I–IV harbored an additional GyrA-87N mutation (Figure 3).

Genomic analysis revealed that clade VI isolates contained some unique characteristics when compared with clades I–V. All of clade VI harbored the plasmid p72_X3_OXA181 with *bla*_OXA-181_ that contained the IncX3 and truncated ColKp3 replicons (Appendix Figure 2). Approximately 30% of isolates in clades I–V contained various carbapenemase genes, namely *bla*_KPC-2_ (n = 184), *bla*_KPC-3_ (n = 4), *bla*_NDM-1_ (n = 3), *bla*_NDM-5_ (n = 2), and *bla*_OXA-48_ (n = 2). KPC-2 was distributed within clades I–V, whereas KPC-3, NDM-1, ΝDΜ-5, and OXA-48 were restricted to clade V (Figure 3). Most ST307 isolates in clades I–VI contained the extended-spectrum β-lactamase gene *bla*_CTX-M-15_ (684/708, 96.6%) that was adjacent to IS*Ecp1* (682/684, 99.7%) situated on a FIB-like plasmid and showed high similarities to the previously described pKPN3–307_type A plasmid (*15*).

We identified 5 prophages (72_phage 1–5) and 1 novel integrative conjugative element (72_ICE) within the 88 sequenced ST307 that belonged to clade VI (Figure 3); the 72_phages 2, 3, and 4 correlated with the previously described ST307 phages 1, 3, and 4 (*15*). The 72_phage1, 72_phage5, and 72_ICE were mainly restricted in clade VI; 72_ICE was unique to clade VI, whereas 72_phage1 was also present in 5 isolates and 72_phage5 in 23 isolates from clade V. The 72_phage3 and 72_phage4 were found in clade VI and a subset of clade V, and the 72_phage 2 was part of ST307 isolates from clusters I–VI (Figure 3).

### Origin of ST307 Clade VI 

**Figure 4:**
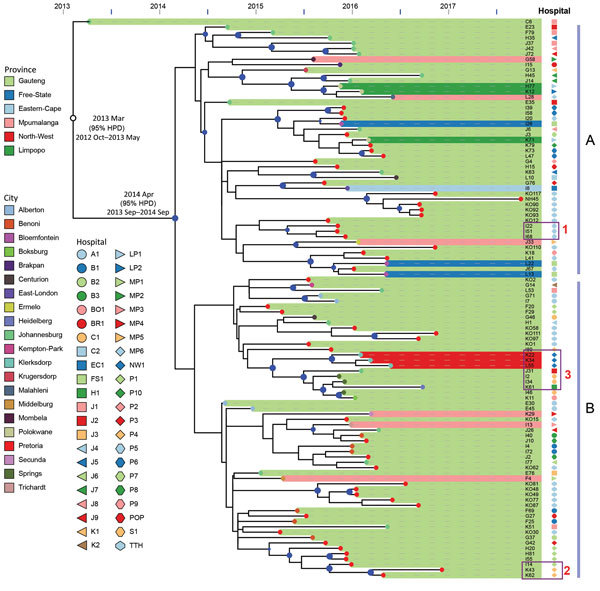
Bayesian evolution analysis of *Klebsiella pneumoniae* sequence type (ST) 307 in South Africa hospitals, January 2014–December 2016. A and B indicate the 2 distinct lineages that evolved within Gauteng Province. Highlighted areas depict the provinces from which isolates were obtained, and colored dots at the tips of areas represent the cities from which the isolates were obtained (e.g., for Gauteng Province, Pretoria is red, Johannesburg is green). Dark blue dots at branch points indicate posterior probability >70%, and dot size is proportional to posterior probability values. Hospital locations are indicated as follows; all are private except for Tshwane tertiary hospital: A1, Alberton; J1–J10, Johannesburg; P1–P8, Pretoria; B1–3, Benoni; BO1, Boksburg; BR1, Brakpan; C1–C2, Centurion; EC1, Eastern Cape Province; FS1, Free State Province; K1, Krugersdorp; K2, Kempton Park; LP1–2, Limpopo Province; MP1–6, Mpumalanga Province; NW1, North West Province; S1, Springs; TTH, Tshwane (tertiary hospital). Box 1 shows intrahospital spread: highly related ST307 isolates were obtained from patient I51 and patient I68 in hospital P5. Box 2 shows interhospital spread: patient I14 was transferred from hospital P1 to hospital P4; 3 months later, highly related ST307 was isolated from patient K43 at hospital P4. Box 3 shows intercity and interprovince spread: patient I2 from Springs, patient K61 from Heidelberg, and patient K22 from North West Province, all with highly related ST307 isolates, had previously received medical care at hospital J2 in Johannesburg. HPD, highest posterior density interval; POP, outpatient.

Bayesian evolution analysis conducted with BEAST estimated the time to most recent common ancestor of clade VI around March 2013, ≈2 months before the first isolate (C6) that was collected in May 2013 from a Johannesburg hospital, J1 (Figure 4). We estimated the mean evolutionary rate as 1.16 × 10^−6^ substitutions/site/year (95% highest posterior density 7.7 × 10^−7^ to 1.6 × 10^−6^ substitutions/site/year), corresponding to 5.7 substitutions/genome/year among clade VI. This analysis placed the outbreak node, from which all outbreak isolates were derived, at April 2014 (95% highest posterior density September 2013–September 2014).

Two distinct lineages evolved within Gauteng Province from the outbreak node. Lineage A originated in Johannesburg and lineage B in Alberton, and they spread to different hospitals across Gauteng (both lineages), Mpumalanga (both lineages), Free State (lineage A), Eastern Cape (lineage A), Limpopo (lineage A), and North West Provinces (lineage B) (Figure 4).

### Dissemination by Movement of Patients

Genomics, combined with admission, discharge, and transfer data, showed evidence of intrahospital spread. For example, patient I51 in hospital P5 was transferred during December 2015 from the medical intensive care unit to the high-risk unit. Subsequently a highly related ST307 was isolated during January 2016 from patient I68 in the high-risk unit (Figure 4). We also observed interhospital spread when patient I14 was transferred from the surgical intensive care unit at hospital P1 to hospital P4 during September 2016. In December 2016, a highly related ST307 was isolated at hospital P4 from patient K43 (Figure 4). Intercity and interprovince spread were illustrated by patient I2 from the city of Springs, patient K61 from the town of Heidelberg, and patient K22 from the North West Province, all with highly related ST307 isolates, having previously received medical care at hospital J2 in Johannesburg (Figure 4).

## Discussion

*K. pneumoniae* producing OXA-181 was first described as isolated in hospitals in India during 2006–2007 (*17*), with a subsequent report from Oman (*18*). OXA-181 has a global distribution, is the second most common OXA-48 derivative, and differs from OXA-48 by 4 amino acid substitutions, while retaining the same spectrum of activity (*3*). Various *K. pneumoniae* clones with OXA-48–like carbapenemases have been described from several localized hospital outbreaks in Africa, Europe, and the Middle East (*3*). Previous studies have shown that *bla*_OXA-181_ are present on ColE2-type (*18*), IncT (*19*), IncN (*20*), and IncX3 (*21*) plasmids.

We describe the dissemination of a carbapenemase-producing clone, *K. pneumoniae* ST307 containing an IncX3 plasmid with *bla*_OXA-181_, across 23 cities and towns within 6 provinces in South Africa. The plasmid (p72_X3_OXA181) was identical to other IncX3 plasmids with *bla*_OXA-181_ previously reported from China (*21*) and Angola (*22*). We tracked (with PCR) and confirmed (with WGS) the presence of ST307 within Gauteng, Mpumalanga, North West, Limpopo, Free State, and Eastern Cape provinces. Genomics combined with admission, discharge, and transfer data showed intrahospital, interhospital, intercity, and interprovince spread to involve 350 patients in 42 private hospitals.

*K. pneumoniae* ST307 is a superclone that emerged during the mid-1990s and was responsible for several worldwide nosocomial outbreaks (*23*). The earliest published report was in 2013 from hospitals in Texas, USA (*24*), and it has since been reported globally (*23*). ST307 is associated with several antimicrobial resistance determinants, including CTX-M-15 (*25*), KPC (*26*), OXA-48 (*27*), NDM-1 (*28*), and *mcr-1* (*29*). Recent reports from Texas (*16*), Colombia (*26*), and Italy (*30*) have shown that ST307 is replacing ST258 as the most prevalent clone associated with multidrug resistance. Certain characteristics of ST307 may lead to increased fitness, persistence, and adaptation to the hospital environment and the human host (*15*).

*K. pneumoniae* ST307 belongs to 6 distinct clades: US clades I–IV; an international clade, V; and South Africa clade VI. The presence of 72_ICE, p72_X3_OXA181 is unique to clade VI and, with other genetic changes, may have played a role in the success of this clade. Bayesian evolution analysis showed that clade VI emerged around March 2013 and evolved during 2014 into 2 distinct lineages that spread across northeastern South Africa over a 15-month period.

In 2014, the World Health Organization reported that key tools to tackle AMR, such as basic surveillance systems to track and monitor the problem, do not exist in many countries (*1*). This study highlighted the public health and clinical utility of using WGS data to develop rapid, reliable, and user-friendly molecular surveillance methods such as PCR for tracking emerging AMR clones and plasmids during outbreaks. We designed PCR primers for tracking ST307 and p72_X3_OXA181 across northeastern South Africa. From a global perspective, this study is an example of a productive collaboration between resource-limited and industrialized countries that rapidly generated cost-effective PCR methodologies to track an emerging AMR clone.

*K. pneumoniae* ST307 clade VI spread rapidly between various private hospitals across South Africa. The reasons for this are unclear, but the medical community needs to know how and why this happened. The increase in OXA-48–like bacteria occurred during a period of high carbapenem usage in the private sector (J. Coetzee, unpub. data). Patients often visit various private hospitals during their treatment. Our results suggest that the intrahospital, interhospital, intercity, and interprovince movements of patients were responsible for the dissemination of ST307.

It is imperative that the medical community continues to explore the reasons for the spread of ST307. Studies investigating the pathogenicity, fitness, adaptiveness, and evolution of ST307 clade VI are currently in progress. The South Africa clade VI has the potential to be introduced to other countries and the ability to cause devastating countrywide outbreaks associated with substantial healthcare costs. A recent report from the United Kingdom highlighted the economic implications associated with a 10-month outbreak of carbapenemase-producing *K. pneumoniae* affecting 40 patients from 5 hospitals across London, costing around €1.1 million (*31*). Clinical studies are also urgently required to investigate the reasons for the high transmission rates of *K. pneumoniae* ST307. Such projects will serve as models to predict what could happen with the continuing emergence of successful clones among clinically relevant bacteria (*32*).

Appendix Additional information about *Klebsiella pneumoniae* ST307 with *bla*_OXA-181,_ South Africa, 2014–2016.
