# Supplementary material for: Klebsiella pneumoniae ST307 with blaOXA-181, South Africa, 2014–2016
Source: Emerg Infect Dis. 2019 Apr;25(4):739–47. doi: 10.3201/eid2504.181482 (PMC6433043; doi:10.3201/eid2504.181482)
Supplement: Appendix — Additional information about Klebsiella pneumoniae ST307 with blaOXA-181, South Africa, 2014–2016. [file 18-1482-Techapp-s1.pdf]

Article DOI: https://doi.org/10.3201/eid2504.181482

Klebsiella pneumoniae ST307 with *bla*$_{OXA-181}$, South Africa, 2014–2016

Appendix

Healthcare Systems in South Africa

The South Africa healthcare system consists of a large public sector, a smaller private sector, and a nongovernmental organizations sector (which focuses mainly on HIV and tuberculosis). The public health system consists of primary healthcare clinics, district hospitals, and academic hospitals (at the tertiary level) where advanced diagnostic procedures and treatments are provided. There are 376 public hospitals in South Africa. The private healthcare system is made up of healthcare professionals who provide their services on a private basis, usually funded by the subscriptions of persons to medical aid schemes. Private healthcare practitioners provide services through private hospitals. There are 238 private hospitals in South Africa.

Ampath Laboratories

Ampath is a large private clinical laboratory that provides pathology-related services to private healthcare practitioners and their patients, especially to those in Gauteng Province (the northern part of the country). Ampath serves ≈50% of the private healthcare market in South Africa. They have an extensive national network of more than 300 facilities that includes reference laboratories, regional laboratories, satellite laboratories and service depots.

The Ampath Molecular Diagnostic Reference Center (Ampath-MDRC) is situated in Pretoria, Gauteng Province, and routinely screens all carbapenem-nonsusceptible *Enterobacteriaceae* (referred from regional laboratories) for carbapenemases using commercial PCR methodology. Due to the nature of the private healthcare system (various hospitals with different healthcare professionals), it was impossible to collect clinical data from patients in that sector.

Selection of Isolates

We selected *K. pneumoniae* isolates that tested positive by PCR for ST307 (n = 88) to represent different geographic locations, time periods, and specimens. The selection criteria were as follows: n = 5 from a private Alberton hospital A1 (July 2014–December 2015); n = 19 from private Johannesburg hospitals J1–J10 (July 2014– December 2016); n = 31 from 10 private Pretoria hospitals P1–P10 (October 2014 – December 2016); n = 6 from 3 private Benoni hospitals B1–B3 (May 2015–January 2016); n = 2 from 1 private Boksburg hospital BO1 (February 2016, July 2016); n = 1 from private Brakpan hospital BR1 (December 2015); n = 2 from 2 private Centurion hospitals C1–C2 (September 2015, June 2016); n = 1 from a private Eastern Cape hospital EC1 (December 2015), n = 3 from a private Free State hospital FS1 (January– July 2016), n = 1 from a private Krugersdorp hospital K1 (November 2015); n = 1 from a private Kempton Park hospital K2 (September 2015), n = 3 from 2 private Limpopo hospitals LP1–2 (December 2015–April 2016), n = 6 from 6 private Mpumalanga hospitals MP1–MP6 (April 2014– July 2016), n = 3 from a private North-West hospital NW1 (May 2016–July 2016), n = 3 from a private Springs hospital S1 (December 2015–March 2016). The sequenced isolates were obtained from the following specimens: urine (42%), respiratory tract (21%), intra-abdominal (17%), blood (16%), and others (4%).

Validation of Primers and Methodologies

We used the following isolates of *K. pneumoniae* with IncF, IncX3, IncN, IncL/M, IncC types of plasmids for validation of the primers for sensitivity and specificity: ST307 (n = 35), ST11 (n = 9), ST14 (n = 5), ST15 (n = 9), ST17 (n = 1), ST29 (n = 1), ST37 (n = 1), ST39 (n = 3), ST47 (n = 1), ST101 (n = 11), ST147 (n = 12), ST218 (n = 1), ST258 (n = 15), ST278 (n = 8), ST336 (n = 2), ST383 (n = 5), ST391 (n = 2), ST392 (n = 2), ST393 (n = 6), ST405 (n = 1), ST423 (n = 2), ST437 (n = 1), ST485 (n = 2), ST525 (n = 2), ST831 (n = 2), ST972 (n = 1), ST1373 (n = 1), and ST1805 (n = 1).

We tested the performance of the PCR methodologies first as a pilot study and then during implementation. When comparing WGS results with PCR results, the ST307, IncX3 and

IS3000-OXA-181 PCR screening displayed 97% correlation with WGS results, furthering the evidence that the PCR approach was highly reliable.

We performed each amplification with 1X JumpStart Taq ReadyMix (Sigma-Aldrich, https://www.sigmaaldrich.com), 0.1 – 0.2 µM for each primer, and 2 µL of DNA in a total volume of 25 µL. Thermocycling was done using Veriti Thermal Cycler (Applied Biosystems, http://www.thermofisher.com/applied/biosystems) under the following conditions:: initial denaturation 95°C for 5 min; 30 cycles of 95°C for 30 s, 55°C for 30 s, and 72°C for 30 s, and a final extension of 72°C for 5 min.

We also screened the isolates for OXA-48-IS1999 using the following primers: OXA-R (Appendix Table 1) and IS1999 (3′-CTTAGAGGCCAGCATCAAGC-5′). Only 1 isolate, I72, was positive for both OXA-181 (amplicon of 724 bp) and OXA-48 (amplicon of 307bp).

Identification and Susceptibility Testing of Bacterial Isolates

We used matrix-assisted laser desorption ionization–time of flight mass spectrometry for identification testing (Vitek AMS; bioMérieux Vitek Systems Inc., https://www.biomerieux-usa.com) and VITEK 2 instrument (bioMérieux Vitek Systems) for susceptibility testing. Susceptibilities to the following drugs were determined: ampicillin (AMP), amoxicillin-clavulanic acid (AMC), piperacillin-tazobactam (TZP), cefotaxime (CTX), ceftazidime (CAZ), imipenem (IPM), meropenem (MER), ertapenem (ERT), amikacin (AMK), gentamicin (GEN), ciprofloxacin (CIP), and trimethoprim-sulfamethoxazole (SXT). Throughout this study, results were interpreted using Clinical Laboratory Standards Institute (CLSI) criteria for broth dilution. Carbapenem susceptibilities (ERT, IPM, and MER), colistin (COL), and tigecycline (TGC) MICs were determined using E-tests (bioMérieux Inc.) according to the manufacturer's instructions. The European Committee for Antimicrobial Susceptibility Testing (EUCAST) breakpoint was used for COL and the FDA breakpoint was used for TGC.

Pulsed-Field Gel Electrophoresis (PFGE)

Genetic relatedness of *K. pneumoniae* isolates was examined by comparing *Xba*I digested profiles generated by PFGE analysis using the standardized *E. coli* (O157:H7) protocol from the Centers for Disease Control and Prevention (CDC), Atlanta, GA, USA (*6*). The subsequent

PFGE analyses were performed on a CHEF-MAPPER XA apparatus (Bio-Rad Laboratories, http://www.Bio-Rad.com). Gel images in tiff format were exported to BioNumerics software version 3.0 (Applied Maths, http://www.applied-maths.com) for analysis. Comparisons were made using the band-based Dice coefficient, which is a binary coefficient measuring similarity based upon common and different bands. Dendrograms were generated using Unweighted Pair Group Method using Arithmetic averages (UPGMA) method with 1.5% position tolerance. DNA relatedness was calculated based on the Dice coefficient and isolates were considered to be genetically related if the Dice coefficient correlation was $\geq$80%, which corresponds to the "possibly related (4–6 bands difference)" criteria of Tenover et al. (*7*). The $bla_{\text{OXA-181}}$ plasmid detection and size estimation was performed using S1-nuclease linearization, followed by PFGE and Southern blot hybridization (*8*).

A dominant pulsotype with >80% similar PFGE profiles (named pulsotype A), was detected among 310/471 (66%) of OXA-48–like isolates (data not shown). Forty isolates exhibited >70% similarity of PFGE profiles to pulsotype A, suggesting that they were related to pulsotype A and were named AR. The remaining isolates were not clonally related, i.e., exhibited <60% similar PFGE profiles and did not show patterns similar to those of pulsotypes A or AR.

Whole-Genome Sequencing and Analysis

The SMRT analysis portal v2.3.0 (Pacific Biosciences, http://www.pacb.com) was used for I72 genome, with raw sequence reads assembled de novo using the HGAP3 protocol, and error correction and polishing with Quiver v1, and closure with Circlator (*1*). Further error correction was performed by mapping Illumina short reads from the same isolate to the PacBio assembly with Pilon v1.22 (*2*). Illumina raw sequencing reads were trimmed using Trimmomatic v0.36 (*3*), followed by de novo assembly, using SPAdes v3.11.1 (*4*) and annotation by Prokka v1.12 (*5*). The multilocus sequence type (MLST) was determined *in silico* using MLST (https://github.com/tseemann/mlst), while the acquired resistance gene and plasmid replicons were determined using Abricate (https://github.com/tseemann/abricate) and ARIBA (*6*) based on ResFinder (*7*) and PlasmidFinder (*8*) databases.

Root-to-tip correlations to test for a temporal signal in the data was performed using TempEST (*9*), and a significant positive slope for the alignment was present ($p<0.001$, $R = 0.527$). BEAST v2.4.7 (*10*) was used to estimate a timed phylogeny with concatenated recombination-free core SNP alignment. The GTR substitution model was selected based on evaluation of substitution models in bModelTest (*11*). BEAST analyses were performed using a coalescent constant population model and a coalescent Bayesian skyline model. In addition, a strict clock, with a lognormal prior, and a relaxed clock (both lognormal and exponential) were tested. Markov chain Monte Carlo (MCMC) was run for 100 million iterations sampling every 10 thousand steps and convergence was checked by inspecting the effective sample sizes and parameter value traces in the software Tracer v1.6.0. The model fit across analyses was compared using Tracer v1.6 (*12*). A relax exponential clock with the Bayesian skyline was selected as the most appropriate model.

PacBio Whole-Genome Sequencing of I72

I72 was identified as *K. pneumoniae* subsp. *pneumoniae* ST307and the chromosomal length was 5.430 Mb. It harbored 3 plasmids, namely p72_FIBkpn containing $\textit{bla}_{\text{CTX-M-15}}$, p72_L/M_OXA48 containing $\textit{bla}_{\text{OXA-48,}}$ and p72_X3_OXA181 containing $\textit{bla}_{\text{OXA-181}}$. p72_X3_OXA181 was 51kb in size, harbored the IncX3 and truncated ColKp3 replicons with $\textit{bla}_{\text{OXA-181}}$ and *qnrS1* (Appendix Figure 2). The p72_FIBkpn plasmid was 193 kb in size, contained FIIK and FIBK replicons and harbored an array of antimicrobial resistance genes, namely $\textit{bla}_{\text{CTX-M-15}}$, $\textit{bla}_{\text{OXA-1}}$, $\textit{bla}_{\text{TEM-1}}$, *aac(6')-Ib-cr, aph(3")-Ib, aph (6)-Id, qnrB1, catB4, strAB, tetA, sul2,* and *dfrA14*. p72_L/M_OXA48 was 64kb in size and contained only 1 antimicrobial resistance gene namely $\textit{bla}_{\text{OXA-48}}$ (Appendix Figure 2).

Identification of the $\textit{bla}_{\text{OXA181}}$ Harboring IncX3 Plasmids

The genome sequences were de novo assembled using plasmidSPAdes (*13*), followed by extraction of the $\textit{bla}_{\text{OXA181}}$-harboring contigs, using blastdbcmd. The $\textit{bla}_{\text{OXA-181}}$ contigs were "BLAST" against PlasmidFinder (*8*) database. The results showed that $\textit{bla}_{\text{OXA-181}}$ co-existed on the same contig as a truncated ColKp3 replicon gene, similar to that of p72_OXA181_X3.The identification of p72_OXA181_X3 were investigated using Bandage assembly graph viewer (*14*)

and BLASTn. The plasmid region was identified by BLASTn searching for the p72_OXA181_X3 “core” genes, excluding insertion or repeated sequences.

Enterobacteriaceae with Carbapenemases other than *K. pneumoniae* from 2014–16

Eighteen *Enterobacter cloacae* complex and 7 *E. coli* with carbapenemases were isolated at Ampath laboratories during 2014–2016. The majority of *E. cloacae* complex was positive for NDMs (n = 15) followed by OXA-48-like (n = 2) and KPC (n = 1); 2 of the *E. coli* was positive for NDM and 2 for OXA-48-like. WGS plasmid mining, S1-PFGE and Southern blotting showed that p72_X3_OXA181 was present among OXA-48-like *Enterobacter cloacae* complex and *E. coli*.

Infection Prevention and Control Measures

Routine auditing of hospital-acquired infections is part of continuous surveillance at the private hospitals. All carbapenem resistant microorganisms (CRE) detected at Ampath and satellite laboratories were communicated to the treating physician and to the infection prevention control (IP&C) practitioners on a daily basis. When an increase of CRE was noted, the affected hospital implemented CDC guidelines for CRE infection prevention and control (*15*). Outbreak response teams were identified within each hospital management during information sharing meetings with management and heads of departments. Infection prevention practitioners held weekly information sessions with all the wards and increased hand hygiene practices. Contact precautions training and education on donning and removal of gloves and aprons were monitored. Environmental cleaning was systematically actively pursued.

All patients within the same wards with newly laboratory confirmed CREs were screened using rectal swabs inoculated on chromID Carba (bioMérieux). All high-risk patients were screened on admission and cohorted immediately if identified. Active surveillance cultures consisted of stool, rectal swabs, ostomy output, wound swabs and endotracheal suctions as requested by the IPC team. Isolation wards were identified at each hospital and patients were cohorted. Strict access control to wards for staff and visitors was implemented. All patients were bathed with 2% chlorohexidine daily and when transferred in or out of the facility.

References

1. Hunt M, Silva ND, Otto TD, Parkhill J, Keane JA, Harris SR. Circlator: automated circularization of genome assemblies using long sequencing reads. Genome Biol. 2015;16:294. PubMed http://dx.doi.org/10.1186/s13059-015-0849-0

2. Walker BJ, Abeel T, Shea T, Priest M, Abouelliel A, Sakthikumar S, et al. Pilon: an integrated tool for comprehensive microbial variant detection and genome assembly improvement. PLoS One. 2014;9:e112963. PubMed http://dx.doi.org/10.1371/journal.pone.0112963

3. Bolger AM, Lohse M, Usadel B. Trimmomatic: a flexible trimmer for Illumina sequence data. Bioinformatics. 2014;30:2114–20. PubMed http://dx.doi.org/10.1093/bioinformatics/btu170

4. Bankevich A, Nurk S, Antipov D, Gurevich AA, Dvorkin M, Kulikov AS, et al. SPAdes: a new genome assembly algorithm and its applications to single-cell sequencing. J Comput Biol. 2012;19:455–77. PubMed http://dx.doi.org/10.1089/cmb.2012.0021

5. Seemann T. Prokka: rapid prokaryotic genome annotation. Bioinformatics. 2014;30:2068–9. PubMed http://dx.doi.org/10.1093/bioinformatics/btu153

6. Hunt M, Mather AE, Sánchez-Busó L, Page AJ, Parkhill J, Keane JA, et al. ARIBA: rapid antimicrobial resistance genotyping directly from sequencing reads. Microb Genom. 2017;3:e000131. PubMed http://dx.doi.org/10.1099/mgen.0.000131

7. Zankari E, Hasman H, Cosentino S, Vestergaard M, Rasmussen S, Lund O, et al. Identification of acquired antimicrobial resistance genes. J Antimicrob Chemother. 2012;67:2640–4. PubMed http://dx.doi.org/10.1093/jac/dks261

8. Carattoli A, Zankari E, García-Fernández A, Voldby Larsen M, Lund O, Villa L, et al. In silico detection and typing of plasmids using PlasmidFinder and plasmid multilocus sequence typing. Antimicrob Agents Chemother. 2014;58:3895–903. PubMed http://dx.doi.org/10.1128/AAC.02412-14

9. Rambaut A, Lam TT, Max Carvalho L, Pybus OG. Exploring the temporal structure of heterochronous sequences using TempEst (formerly Path-O-Gen). Virus Evolution. 2016;2:vew007.

10. Bouckaert R, Heled J, Kühnert D, Vaughan T, Wu CH, Xie D, et al. BEAST 2: a software platform for Bayesian evolutionary analysis. PLOS Comput Biol. 2014;10:e1003537. PubMed http://dx.doi.org/10.1371/journal.pcbi.1003537

11. Bouckaert RR, Drummond AJ. bModelTest: Bayesian phylogenetic site model averaging and model comparison. BMC Evol Biol. 2017;17:42. PubMed http://dx.doi.org/10.1186/s12862-017-0890-6

12. Baele G, Lemey P, Bedford T, Rambaut A, Suchard MA, Alekseyenko AV. Improving the accuracy of demographic and molecular clock model comparison while accommodating phylogenetic uncertainty. Mol Biol Evol. 2012;29:2157–67. PubMed http://dx.doi.org/10.1093/molbev/mss084

13. Antipov D, Hartwick N, Shen M, Raiko M, Lapidus A, Pevzner PA. plasmidSPAdes: assembling plasmids from whole genome sequencing data. Bioinformatics. 2016;32:3380–7. PubMed

14. Wick RR, Schultz MB, Zobel J, Holt KE. Bandage: interactive visualization of de novo genome assemblies. Bioinformatics. 2015;31:3350–2.

15. Centers for Disease Control and Prevention. Guidance for the control of carbapenem-resistant *Enterobacteriaceae* (CRE): 2012 CRE Toolkit. 2012. [cited 2019 Feb 8]. http://www.safecarecampaign.org/assets/cre-toolkit_final_6_18_20122.pdf

Appendix Table 1. PCR primers and amplification conditions

Primers	Sequence	Target	Amplicon size, bp	Sensitivity	Specificity
307-F2	3′-AGGAAAGTCGCCGCAGGAGGAT-5′	ST307*	628	97%	94%
307-R2	3′-TGCTGCTGCCATAAAACGCACCT-5′				
X3-F1	3′-TGCAGCTCTGAAAGAGGGAGCTAA-5′	IncX3	556	99%	96%
X3-R1	3′-TCTAAGCATGTGAGCCATTCTCCCT-5′				
IS3000-F	3′-TTTACCCGTCAGCGAAAAGT-5′	IS3000-OXA	724	98%	95%
OXA-R	3′-TTTCTTGCCATTCCTTTGCT-5′				

*target; 72_phage 2.

Appendix Table 2. Carbapenemase-producing *Klebsiella pneumoniae* isolates identified in South Africa, January 2014–December 2016

Strain	Province	Jan–Jun 2014	Jul–Dec 2014	Jan–Jun 2015	Jul–Dec 2015	Jan–Jun 2016	Jul–Dec 2016
KPC (n = 10)		1	0	3	1	5	0
Alberton	Gauteng						
Pretoria	Gauteng	1		1			
East London	East-Cape			2			
Johannesburg	Gauteng				1	3	
Roodepoort	Gauteng					1	
Krugersdorp	Gauteng					1	
NDM (n = 58)		7	19	8	11	11	2
Pretoria	Gauteng		6	2	4	2	1
Johannesburg	Gauteng	6	10	5	4	5	
Alberton	Gauteng				2		
Richards Bay	KwaZulu	1					
Bloemfontein	Free State		3	1	1	2	1
Trichardt	Mpumalanga					1	
Mbombela	Mpumalanga					1	
VIM (n = 35)		7	12	6	8	1	1
Emalahleni	Mpumalanga	1	2		1		
Pretoria	Gauteng	2	6	2	5		1
Johannesburg	Gauteng	4	3	3	2	1	
Bloemfontein	Free State		1				
Springs	Gauteng			1			
OXA-48–like (n = 471)		21	33	41	162	187	27
Johannesburg	Gauteng	15	18	16	54	63	7
Cape Town	Cape		1		7	2	
Alberton	Gauteng	2	8	3	11	7	1
Heidelberg	Gauteng	1				2	1
Krugersdorp	Gauteng	1			2	1	
Pretoria	Gauteng	1	3	16	63	69	14
Somerset	Cape	1					
Springs	Gauteng		2	1	2	3	
Tzaneen	Limpopo		1				
Benoni	Gauteng			4	13	9	1
Middelburg	Mpumalanga			1	2	1	
Brakpan	Gauteng				1	2	
Centurion	Gauteng				1	2	
East London	East Cape				1	1	
Kempton	Gauteng				1	2	
Mbombela	Mpumalanga				1	4	
Polokwane	Limpopo				1	3	
Trichardt	Mpumalanga				1	1	
VDBijlPark	Gauteng				1		
Bloemfontein	Free State					3	
Boksburg	Gauteng					4	
Ermelo	Mpumalanga					1	
Klerksdorp	North West					5	1
Secunda	Mpumalanga					2	1
Emalahleni	Mpumalanga					3	1

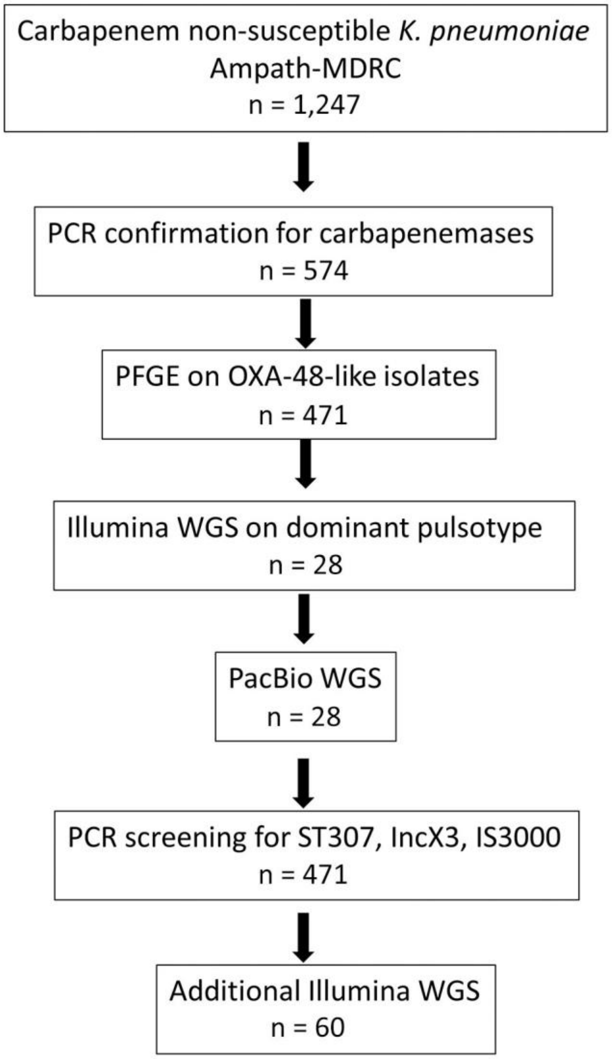

Appendix Figure 1. Workflow for study of *Klebsiella pneumoniae* ST307 with *bla*$_{OXA-181}$, South Africa, 2014–2016.

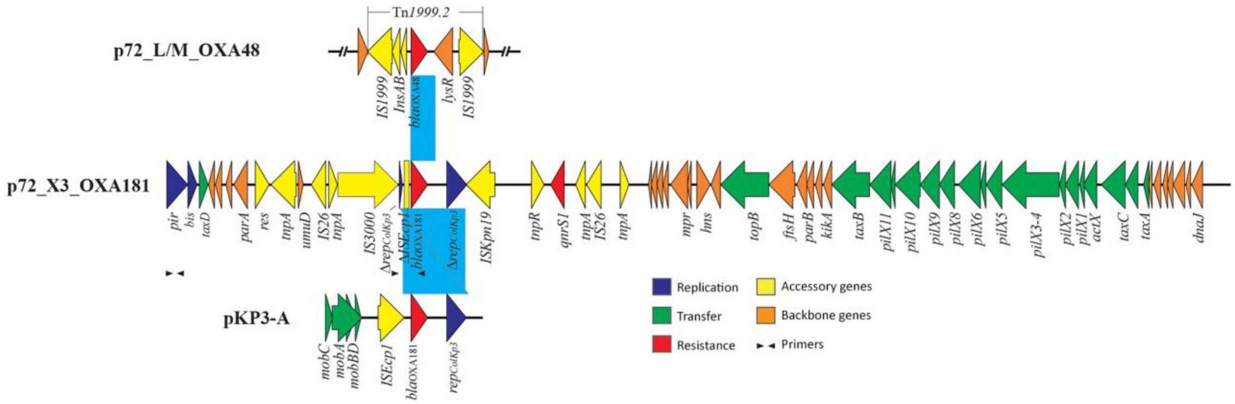

Appendix Figure 2. Sequence homology of p72_X3_OXA181 containing *bla*$_{OXA-181}$ from study of *Klebsiella pneumoniae* ST307, South Africa, 2014–2016.
